# Di-μ-*tert*-butanolato-bis­[bis­(η^5^-cyclo­penta­dien­yl)erbium(III)]

**DOI:** 10.1107/S1600536808004248

**Published:** 2008-02-15

**Authors:** Sandro Pagano, Wolfgang Schnick

**Affiliations:** a Department Chemie und Biochemie, Ludwig-Maximilians-Universität München, Lehrstuhl für Anorganische Festkörperchemie, Butenandtstrasse 5–13 (D), D-81377 München, Germany

## Abstract

In the centrosymmetric title compound, [Er_2_(C_5_H_5_)_4_(C_4_H_9_O)_2_], each Er atom is in a distorted tetra­hedral coordination environment, coordinated by two cyclo­penta­dienyl rings and two *tert*-but­oxy groups, forming a dimeric complex bridged through the *tert*-but­oxy groups.

## Related literature

During our search for highly reactive mol­ecular precursors, we characterized a series of lanthanide amide (Baisch, Pagano, Zeuner, Barros *et al.*, 2006 ▶) and carbamate complexes (Baisch, Pagano, Zeuner & Schnick, 2006 ▶). The synthesis of [Er_2_{μ-η^1^:η^2^-OC(OBu^*t*^)NH}Cp_4_] and its application as a precursor is described by Zeuner *et al.* (2008 ▶). For related literature and a general overview of cyclo­penta­dienyl-containing compounds of the lanthanides, see: Schumann *et al.* (1995 ▶).
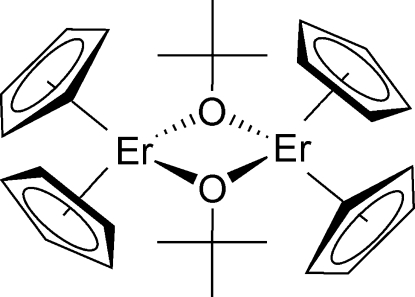


## Experimental

### 

#### Crystal data

[Er_2_(C_5_H_5_)_4_(C_4_H_9_O)_2_]
*M* *_r_* = 741.10
Monoclinic, 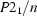

*a* = 8.3905 (17) Å
*b* = 15.628 (3) Å
*c* = 9.950 (2) Å
β = 101.85 (3)°
*V* = 1276.9 (5) Å^3^
*Z* = 2
Mo *K*α radiation
μ = 6.55 mm^−1^
*T* = 200 (2) K
0.13 × 0.07 × 0.04 mm

#### Data collection

Nonius KappaCCD diffractometer
Absorption correction: multi-scan (*SADABS*; Sheldrick, 2001 ▶) *T*_min_ = 0.594, *T*_max_ = 0.783
5666 measured reflections
2912 independent reflections
2578 reflections with *I* > \2s(*I*)
*R*_int_ = 0.021

#### Refinement

*R*[*F* ^2^ > 2σ(*F* ^2^)] = 0.022
*wR*(*F* ^2^) = 0.053
*S* = 1.05
2912 reflections
149 parameters
H-atom parameters constrained
Δρ_max_ = 1.29 e Å^−3^
Δρ_min_ = −1.23 e Å^−3^

### 

Data collection: *COLLECT* (Nonius, 2004 ▶); cell refinement: *DENZO*/*SCALEPACK* (Otwinowski & Minor 1997 ▶); data reduction: *DENZO*/*SCALEPACK*; program(s) used to solve structure: *SHELXS97* (Sheldrick, 2008 ▶); program(s) used to refine structure: *SHELXL97* (Sheldrick, 2008 ▶); molecular graphics: *DIAMOND* (Brandenburg 1999 ▶); software used to prepare material for publication: *SHELXL97*.

## Supplementary Material

Crystal structure: contains datablocks I, global. DOI: 10.1107/S1600536808004248/su2044sup1.cif

Structure factors: contains datablocks I. DOI: 10.1107/S1600536808004248/su2044Isup2.hkl

Additional supplementary materials:  crystallographic information; 3D view; checkCIF report

## Figures and Tables

**Fig. 1.**
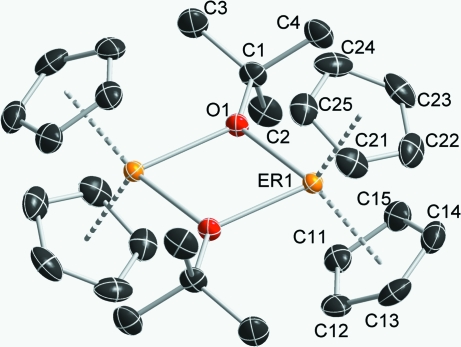
A view of the title compound showing the atom-labelling scheme. Displacement ellipsoids are drawn at the 50% probability level. H– atoms are omitted for clarity.

**Fig. 2.**
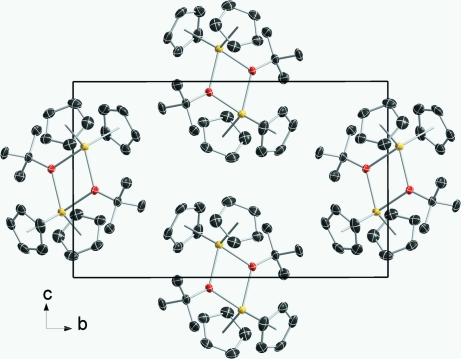
Crystal packing diagram of the title compound viewed along the a axis.

## supplementary crystallographic information

### Comment

By stirring [Er_2_{µ-η^1^:η^2^-OC(OBu*^t^*)NH}Cp_4_] in THF the carbamato moiety decomposes to [Cp_2_Er*^t^*BuO]_2_ and an amorphous solid showing strong C=N vibrations (2190 cm^-1^) in the IR spectra. It is likely that the *tert*-butylcarbamato ligand splits up into *tert*-butanol and cyanate. Attempts to isolate a crystalline cyanato complex were unsuccessful as yet. The structure of [Cp_2_Er*^t^*BuO]_2_ is in accordance with a series of lanthanide-cyclopentadienyl-alcoholate-complexes synthesized so far (Schumann *et al.*, 1995).

### Experimental

[Er_2_{µ-η^1^:η^2^-OC(OBu*^t^*)NH}Cp_4_] was dissolved in dry THF and stirred for 12 h at 297 K. After evaporation of the solvent the orange residue was suspended in dry hexane. The filtrate was stored at 279 K to afford [Cp_2_Er*^t^*BuO]_2_ as pink crystals.

### Refinement

The H atoms were positioned geometrically and refined using a riding model: C(aromatic)–H = 0.95 Å with *U*_iso_(H) = 1.2*U*_eq_(C), and C(aliphatic)–H = 0.98 Å with *U*_iso_(H) = 1.5*U*_eq_(C).

### Crystal data

**Table:**

[Er_2_(C_5_H_5_)_4_(C_4_H_9_O)_2_]	*F*_000_ = 716
*M**_r_* = 741.10	*D*_x_ = 1.928 Mg m^−^^3^
Monoclinic, *P*2_1_/*n*	Melting point: not measured K
Hall symbol: -P 2yn	Mo *K*α radiation λ = 0.71073 Å
*a* = 8.3905 (17) Å	Cell parameters from 14322 reflections
*b* = 15.628 (3) Å	θ = 3.1–27.5º
*c* = 9.950 (2) Å	µ = 6.55 mm^−^^1^
β = 101.85 (3)º	*T* = 200 (2) K
*V* = 1276.9 (5) Å^3^	Block, pink
*Z* = 2	0.13 × 0.07 × 0.04 mm

### Data collection

**Table:**

Nonius KappaCCD diffractometer	2912 independent reflections
Radiation source: fine-focus sealed tube	2578 reflections with *I* > \2s(*I*)
Monochromator: graphite	*R*_int_ = 0.021
Detector resolution: 9 pixels mm^-1^	θ_max_ = 27.5º
*T* = 200(2) K	θ_min_ = 3.2º
φ and ω scans	*h* = −10→10
Absorption correction: multi-scan(SADABS; Sheldrick, 2001)	*k* = −20→20
*T*_min_ = 0.594, *T*_max_ = 0.783	*l* = −12→12
5666 measured reflections	

### Refinement

**Table:**

Refinement on *F*^2^	Hydrogen site location: inferred from neighbouring sites
Least-squares matrix: full	H-atom parameters constrained
*R*[*F*^2^ > 2σ(*F*^2^)] = 0.022	*w* = 1/[σ^2^(*F*_o_^2^) + (0.025*P*)^2^ + 1.7644*P*] where *P* = (*F*_o_^2^ + 2*F*_c_^2^)/3
*wR*(*F*^2^) = 0.053	(Δ/σ)_max_ = 0.002
*S* = 1.05	Δρ_max_ = 1.29 e Å^−^^3^
2912 reflections	Δρ_min_ = −1.23 e Å^−^^3^
149 parameters	Extinction correction: (SHELXL97; Sheldrick, 2008), Fc^*^=kFc[1+0.001xFc^2^λ^3^/sin(2θ)]^-1/4^
Primary atom site location: structure-invariant direct methods	Extinction coefficient: 0.0022 (2)
Secondary atom site location: difference Fourier map	

### Special details

**Table:**

Geometry. All e.s.d.'s (except the e.s.d. in the dihedral angle between two l.s. planes) are estimated using the full covariance matrix. The cell e.s.d.'s are taken into account individually in the estimation of e.s.d.'s in distances, angles and torsion angles; correlations between e.s.d.'s in cell parameters are only used when they are defined by crystal symmetry. An approximate (isotropic) treatment of cell e.s.d.'s is used for estimating e.s.d.'s involving l.s. planes.
Refinement. Refinement of *F*^2^ against ALL reflections. The weighted *R*-factor *wR* and goodness of fit *S* are based on *F*^2^, conventional *R*-factors *R* are based on *F*, with *F* set to zero for negative *F*^2^. The threshold expression of *F*^2^ > σ(*F*^2^) is used only for calculating *R*-factors(gt) *etc*. and is not relevant to the choice of reflections for refinement. *R*-factors based on *F*^2^ are statistically about twice as large as those based on *F*, and *R*- factors based on ALL data will be even larger.

### Fractional atomic coordinates and isotropic or equivalent isotropic displacement parameters (Å^2^)

**Table:**

	*x*	*y*	*z*	*U*_iso_*/*U*_eq_	
Er1	0.080747 (15)	0.036095 (8)	0.666673 (13)	0.01748 (7)	
C15	0.2668 (4)	−0.0762 (3)	0.8373 (4)	0.0332 (8)	
H15	0.2131	−0.1176	0.8821	0.040*	
C13	0.3898 (5)	0.0464 (3)	0.7888 (5)	0.0385 (9)	
H13	0.4331	0.1028	0.7953	0.046*	
C23	−0.0655 (5)	0.0983 (3)	0.8573 (4)	0.0396 (10)	
H23	−0.0683	0.0605	0.9315	0.048*	
C25	−0.1323 (5)	0.1661 (2)	0.6539 (4)	0.0328 (8)	
H25	−0.1887	0.1835	0.5653	0.039*	
C21	0.0166 (5)	0.1986 (2)	0.7245 (4)	0.0349 (8)	
H21	0.0797	0.2414	0.6919	0.042*	
C11	0.3101 (4)	−0.0858 (3)	0.7084 (4)	0.0312 (8)	
H11	0.2906	−0.1350	0.6513	0.037*	
C22	0.0565 (5)	0.1574 (3)	0.8508 (4)	0.0376 (9)	
H22	0.1505	0.1679	0.9203	0.045*	
C14	0.3175 (5)	0.0055 (3)	0.8874 (4)	0.0391 (9)	
H14	0.3053	0.0290	0.9728	0.047*	
C12	0.3865 (4)	−0.0107 (3)	0.6790 (4)	0.0339 (8)	
H12	0.4288	−0.0001	0.5990	0.041*	
C24	−0.1835 (5)	0.1041 (3)	0.7358 (4)	0.0375 (9)	
H24	−0.2809	0.0715	0.7134	0.045*	
O1	0.0788 (3)	0.06891 (14)	0.4454 (2)	0.0195 (4)	
C1	0.1512 (4)	0.1414 (2)	0.3870 (3)	0.0246 (7)	
C2	0.0347 (5)	0.2174 (2)	0.3640 (4)	0.0343 (8)	
H2A	−0.0665	0.2007	0.3014	0.051*	
H2B	0.0853	0.2649	0.3238	0.051*	
H2C	0.0106	0.2354	0.4520	0.051*	
C4	0.1903 (5)	0.1155 (2)	0.2497 (4)	0.0328 (8)	
H4A	0.2719	0.0698	0.2642	0.049*	
H4B	0.2331	0.1651	0.2082	0.049*	
H4C	0.0910	0.0951	0.1883	0.049*	
C3	0.3083 (5)	0.1691 (3)	0.4829 (4)	0.0360 (9)	
H3A	0.2863	0.1820	0.5737	0.054*	
H3B	0.3512	0.2203	0.4457	0.054*	
H3C	0.3886	0.1228	0.4908	0.054*	

### Atomic displacement parameters (Å^2^)

**Table:**

	*U*^11^	*U*^22^	*U*^33^	*U*^12^	*U*^13^	*U*^23^
Er1	0.01723 (10)	0.01828 (10)	0.01704 (10)	0.00043 (5)	0.00380 (6)	−0.00140 (5)
C15	0.0310 (19)	0.038 (2)	0.0284 (19)	0.0078 (16)	0.0010 (14)	0.0104 (16)
C13	0.0205 (18)	0.041 (2)	0.049 (2)	−0.0049 (15)	−0.0040 (16)	0.0010 (19)
C23	0.058 (3)	0.035 (2)	0.033 (2)	0.0116 (19)	0.0257 (19)	0.0020 (17)
C25	0.038 (2)	0.0274 (19)	0.033 (2)	0.0108 (16)	0.0053 (15)	−0.0065 (15)
C21	0.045 (2)	0.0218 (18)	0.042 (2)	−0.0023 (16)	0.0192 (17)	−0.0102 (16)
C11	0.0268 (18)	0.036 (2)	0.0295 (19)	0.0109 (15)	0.0022 (14)	0.0029 (15)
C22	0.042 (2)	0.038 (2)	0.031 (2)	0.0075 (17)	0.0030 (16)	−0.0179 (17)
C14	0.031 (2)	0.054 (3)	0.0276 (19)	0.0070 (19)	−0.0043 (15)	−0.0051 (19)
C12	0.0188 (17)	0.049 (2)	0.035 (2)	0.0079 (16)	0.0079 (14)	0.0053 (18)
C24	0.0297 (19)	0.030 (2)	0.058 (3)	0.0028 (16)	0.0221 (18)	−0.0096 (19)
O1	0.0228 (11)	0.0165 (11)	0.0196 (11)	−0.0040 (9)	0.0050 (8)	0.0020 (9)
C1	0.0294 (17)	0.0178 (16)	0.0278 (17)	−0.0054 (13)	0.0088 (13)	0.0026 (13)
C2	0.045 (2)	0.0182 (17)	0.042 (2)	−0.0002 (16)	0.0144 (17)	0.0095 (16)
C4	0.042 (2)	0.0291 (19)	0.0300 (18)	−0.0063 (16)	0.0147 (15)	0.0041 (16)
C3	0.040 (2)	0.033 (2)	0.036 (2)	−0.0147 (17)	0.0101 (16)	−0.0002 (17)

### Geometric parameters (Å, °)

**Table:**

Er1—O1	2.257 (2)	C25—H25	0.9500
Er1—O1^i^	2.262 (2)	C21—C22	1.390 (6)
Er1—C13	2.633 (4)	C21—H21	0.9500
Er1—C12	2.646 (3)	C11—C12	1.397 (6)
Er1—C23	2.646 (4)	C11—H11	0.9500
Er1—C24	2.673 (3)	C22—H22	0.9500
Er1—C22	2.673 (4)	C14—H14	0.9500
Er1—C11	2.679 (4)	C12—H12	0.9500
Er1—C21	2.682 (4)	C24—H24	0.9500
Er1—C14	2.685 (4)	O1—C1	1.461 (4)
Er1—C25	2.692 (4)	O1—Er1^i^	2.262 (2)
Er1—C15	2.705 (4)	C1—C3	1.523 (5)
C15—C14	1.405 (6)	C1—C4	1.524 (5)
C15—C11	1.411 (5)	C1—C2	1.525 (5)
C15—H15	0.9500	C2—H2A	0.9800
C13—C12	1.406 (6)	C2—H2B	0.9800
C13—C14	1.409 (6)	C2—H2C	0.9800
C13—H13	0.9500	C4—H4A	0.9800
C23—C22	1.391 (6)	C4—H4B	0.9800
C23—C24	1.399 (6)	C4—H4C	0.9800
C23—H23	0.9500	C3—H3A	0.9800
C25—C24	1.391 (6)	C3—H3B	0.9800
C25—C21	1.397 (5)	C3—H3C	0.9800
			
O1—Er1—O1^i^	78.39 (8)	Er1—C13—H13	114.5
O1—Er1—C13	104.21 (12)	C22—C23—C24	108.3 (4)
O1^i^—Er1—C13	134.35 (11)	C22—C23—Er1	75.9 (2)
O1—Er1—C12	85.46 (10)	C24—C23—Er1	75.8 (2)
O1^i^—Er1—C12	107.00 (11)	C22—C23—H23	125.8
C13—Er1—C12	30.90 (13)	C24—C23—H23	125.8
O1—Er1—C23	134.74 (11)	Er1—C23—H23	114.7
O1^i^—Er1—C23	107.78 (11)	C24—C25—C21	108.1 (4)
C13—Er1—C23	101.80 (14)	C24—C25—Er1	74.2 (2)
C12—Er1—C23	131.01 (13)	C21—C25—Er1	74.6 (2)
O1—Er1—C24	108.69 (11)	C24—C25—H25	126.0
O1^i^—Er1—C24	88.69 (11)	C21—C25—H25	126.0
C13—Er1—C24	130.34 (14)	Er1—C25—H25	117.3
C12—Er1—C24	160.99 (13)	C22—C21—C25	108.2 (4)
C23—Er1—C24	30.49 (13)	C22—C21—Er1	74.6 (2)
O1—Er1—C22	121.39 (11)	C25—C21—Er1	75.3 (2)
O1^i^—Er1—C22	137.07 (11)	C22—C21—H21	125.9
C13—Er1—C22	81.17 (13)	C25—C21—H21	125.9
C12—Er1—C22	111.94 (13)	Er1—C21—H21	116.3
C23—Er1—C22	30.31 (13)	C12—C11—C15	108.5 (4)
C24—Er1—C22	50.05 (13)	C12—C11—Er1	73.5 (2)
O1—Er1—C11	100.02 (10)	C15—C11—Er1	75.8 (2)
O1^i^—Er1—C11	83.92 (11)	C12—C11—H11	125.8
C13—Er1—C11	50.50 (13)	C15—C11—H11	125.8
C12—Er1—C11	30.41 (12)	Er1—C11—H11	116.9
C23—Er1—C11	125.04 (12)	C21—C22—C23	107.8 (4)
C24—Er1—C11	148.22 (12)	C21—C22—Er1	75.3 (2)
C22—Er1—C11	123.46 (12)	C23—C22—Er1	73.8 (2)
O1—Er1—C21	91.86 (10)	C21—C22—H22	126.1
O1^i^—Er1—C21	131.93 (10)	C23—C22—H22	126.1
C13—Er1—C21	93.72 (13)	Er1—C22—H22	116.9
C12—Er1—C21	119.13 (13)	C15—C14—C13	107.9 (4)
C23—Er1—C21	49.88 (12)	C15—C14—Er1	75.7 (2)
C24—Er1—C21	49.84 (12)	C13—C14—Er1	72.6 (2)
C22—Er1—C21	30.08 (12)	C15—C14—H14	126.0
C11—Er1—C21	144.01 (12)	C13—C14—H14	126.0
O1—Er1—C14	133.98 (11)	Er1—C14—H14	117.7
O1^i^—Er1—C14	122.53 (12)	C11—C12—C13	107.9 (3)
C13—Er1—C14	30.70 (14)	C11—C12—Er1	76.1 (2)
C12—Er1—C14	50.61 (12)	C13—C12—Er1	74.0 (2)
C23—Er1—C14	81.56 (13)	C12—C12—H12	126.1
C24—Er1—C14	111.95 (13)	C13—C12—H12	126.1
C22—Er1—C14	73.34 (13)	Er1—C12—H12	116.0
C11—Er1—C14	50.14 (13)	C25—C24—C23	107.6 (4)
C21—Er1—C14	98.07 (13)	C25—C24—Er1	75.8 (2)
O1—Er1—C25	84.86 (10)	C23—C24—Er1	73.7 (2)
O1^i^—Er1—C25	101.80 (10)	C25—C24—H24	126.2
C13—Er1—C25	123.85 (13)	C23—C24—H24	126.2
C12—Er1—C25	147.00 (13)	Er1—C24—H24	116.5
C23—Er1—C25	49.88 (12)	C1—O1—Er1	130.07 (19)
C24—Er1—C25	30.06 (12)	C1—O1—Er1^i^	128.22 (18)
C22—Er1—C25	49.74 (12)	Er1—O1—Er1^i^	101.61 (8)
C11—Er1—C25	173.19 (12)	O1—C1—C3	110.4 (3)
C21—Er1—C25	30.13 (12)	O1—C1—C4	109.6 (3)
C14—Er1—C25	123.08 (13)	C3—C1—C4	108.6 (3)
O1—Er1—C15	130.36 (10)	O1—C1—C2	110.9 (3)
O1^i^—Er1—C15	92.60 (11)	C3—C1—C2	108.6 (3)
C13—Er1—C15	50.45 (13)	C4—C1—C2	108.7 (3)
C12—Er1—C15	50.39 (12)	C1—C2—H2A	109.5
C23—Er1—C15	94.67 (12)	C1—C2—H2B	109.5
C24—Er1—C15	119.92 (13)	H2A—C2—H2B	109.5
C22—Er1—C15	98.37 (12)	C1—C2—H2C	109.5
C11—Er1—C15	30.38 (11)	H2A—C2—H2C	109.5
C21—Er1—C15	126.53 (12)	H2B—C2—H2C	109.5
C14—Er1—C15	30.22 (14)	C1—C4—H4A	109.5
C25—Er1—C15	144.28 (12)	C1—C4—H4B	109.5
C14—C15—C11	107.6 (4)	H4A—C4—H4B	109.5
C14—C15—Er1	74.1 (2)	C1—C4—H4C	109.5
C11—C15—Er1	73.8 (2)	H4A—C4—H4C	109.5
C14—C15—H15	126.2	H4B—C4—H4C	109.5
C11—C15—H15	126.2	C1—C3—H3A	109.5
Er1—C15—H15	117.9	C1—C3—H3B	109.5
C12—C13—C14	108.1 (4)	H3A—C3—H3B	109.5
C12—C13—Er1	75.1 (2)	C1—C3—H3C	109.5
C14—C13—Er1	76.7 (2)	H3A—C3—H3C	109.5
C12—C13—H13	125.9	H3B—C3—H3C	109.5
C14—C13—H13	125.9		

Symmetry codes: (i) −*x*, −*y*, −*z*+1.
